# TNPO2 operates downstream of DYNC1I1 and promotes gastric cancer cell proliferation and inhibits apoptosis

**DOI:** 10.1002/cam4.2582

**Published:** 2019-10-11

**Authors:** Libao Gong, Ti Wen, Zhi Li, Yizhe Wang, Jin Wang, Xiaofang Che, Yunpeng Liu, Xiujuan Qu

**Affiliations:** ^1^ Department of Medical Oncology The First Hospital of China Medical University Shenyang China; ^2^ Key Laboratory of Anticancer Drugs and Biotherapy of Liaoning Province The First Hospital of China Medical University Shenyang China

**Keywords:** DYNC1I1, gastric cancer, P300, proliferation, SP1, TNPO2

## Abstract

The import of proteins into the nucleus plays an important role in tumor development. In addition to the classical nuclear import proteins importin‐β and importin‐α, there are many nonclassical nuclear import proteins that include TNPO2. The role of TNPO2 as a nonclassical nuclear import protein in tumors is limited. Our previous studies have shown that DYNC1I1 is a poor prognostic factor for gastric cancer and can promote the proliferation and metastasis of gastric cancer cells. An expression profile chip showed that TNPO2 was its potential downstream. DYNC1I1 upregulated TNPO2 expression by upregulating SP1, following which, SP1 recruited and bound to the P300‐acetylated TNPO2 promoter region histones, and thus promoted TNPO2 expression. At the same time, TNPO2 promoted gastric cancer cell proliferation and inhibited apoptosis by a mechanism that might be depending on the functional expression of P21.

## Graphical abstract

TNPO2 promotes gastric cancer cell proliferation and inhibits apoptosis by upregulating P21. DYNC1I1 upregulates TNPO2 expression by upregulating transcription factor SP1

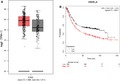

## 1 INTRODUCTION

Gastric carcinoma is one of the most common malignancies, with a relatively high rate of mortality across the world.^1^ In addition, due to lack of effective and predictive capabilities for early screening, gastric cancer in most of the patients has been metastasized by the time of their initial diagnosis. Furthermore, there is currently no particular effective therapeutic target for gastric cancer. Therefore, it is urgent and imperative to explore the molecular mechanism of gastric cancer development.

According to our previous experiments, cytoplasmic dynein intermediate chain 1 (DYNC1I1) is associated with poor prognosis of gastric cancer and promoted proliferation and migration of gastric cancer cells both in vivo and in vitro.^2^ To study the specific underlying mechanism involved in the functional role of DYNC1I1 in gastric cancer, an affymetrix‐mediated scanning microarray of genome‐wide expression profiles after knockdown of DYNC1I1 was conducted. Transportin 2 (TNPO2) is identified as the most prominent gene in downregulating transcriptional expression.

TNPO2 belongs to β‐karyopherin family, which involves a family of factors that can be divided into importins and exportins.^3^, ^4^ The major family members included in it are importin‐β and importin‐α. The cargo proteins are mostly transported by importin‐β into the nucleus, and often use importin‐α as an adaptor. Importins bind to nuclear localization signal (NLS) of the cargo protein in the cytoplasm in many different ways. The complex then passes through the nuclear pore complex (NPC) by interaction with imported protein and nucleoporin.^5^, ^6^ Importins are bound by Ran‐GTP, releasing the cargo proteins in the nucleus and recycling them in the cytoplasm. According to the recent research, karyopherin‐β family is regarded as a potential target for cancer therapy.^3^, ^7^, ^8^, ^9^, ^10^ The TNPO2‐mediated transport of the material into the nuclear process is similar to the above described system. But TNPO2 only transports limited nuclear‐destined cargoes, and participates only in the nuclear process, without participating in the cargo export process.^11^

TNPO2 is a nuclear accessory protein, in which its transport‐assisted protein belongs to the class of RNA‐binding proteins, including HuR and hnRNP. This in turn belongs to noncanonical nuclear pathway^11^, ^12^ that promotes muscle maturation and differentiation. At the same time, it also plays an important role in tumor cells. During apoptosis, TNPO2 inhibits apoptosis in Hela cells.^13^ Recent studies have demonstrated that TNPO2 is highly expressed in malignant pleural mesothelioma (MPM) tissues and cells, which might represent a new cancer gene.^14^ However, the role played by TNPO2 in gastric cancer has not been previously elucidated or reported. As far as we know, there is no report till date on the regulation of TNPO2, and there is very little research on the mechanism of action of it in tumors currently.

Hence, in this study, we initially found that the downregulation of TNPO2 mRNA expression was most significantly observed after silencing DYNC1I1 in gastric cancer cells by gene expression microarray analysis, which indicated that DYNC1I1 as a potential upstream signaling mediator of TNPO2. In addition, the putative mechanism by which DYNC1I1 regulates TNPO2 was explored. The results showed that the TNPO2 transcription factor SP1 plays an important role, as it not only serves as a transcription factor but also simultaneously recruits the P300‐acetylated TNPO2 promoter region histone. Furthermore, the role of TNPO2 in gastric cancer cells was explored, revealing TNPO2 in the promotion of proliferation of gastric cancer cells, and inhibition of apoptosis. It is suggested that the potential downstream mediator of TNPO2 might be P21 under these settings.

## 2 MATERIALS AND METHODS

### 2.1 Cell lines and culture condition

HGC‐27, SGC‐7901, SNU‐216, and MGC‐803 gastric cancer cells were obtained from the Type Culture Collection of the Chinese Academy of Sciences (China). Cells were cultured in RPMI1640 (Gibco) supplemented with 10% fetal bovine serum, 100 U/ml penicillin, and 100 µg/mL streptomycin, and maintained in a 5% CO_2_ incubator at 37°C. Experiments were carried out using logarithmic growth phase cells.

### 2.2 Total RNA isolation and RT‐qPCR

The TRIzol kit was used to extract the total RNA, then the RNA reversely transcribed into cDNA according to the PrimeScript® RT reagent Kit with gDNA Eraser (Takara) kit protocol. After the complementary Deoxyribose Nucleic Acid (cDNA) was amplified, RT‐PCR was used to detect the relative expressions of related genes using SYBR Premix Ex Taq II kit. Primers used in the study were: TNPO2 F: 5′‐CTTTAGGGTGTCTTCCGCGA‐3′, R: 5′‐GAAGACAGTAGGGCACCGTT‐3′; SP1 F: 5′‐CCCTTGAGCTTGTCCCTCAG‐3′, R: 5′‐TGAAAAGGCACCACCACCAT‐3′; GAPDH F: 5′‐GGTGTGAACCATGAGAAGTATGA‐3′, R: 5′‐GAGTCCTTCCACGATACCAAAG‐3′; the relative mRNA expression of TNPO2 and SP1 were estimated by ΔΔCt and normalized to GAPDH.

### 2.3 Western blot analysis

The cells were treated according to different requirements of the experiments then cells were collected using lysis buffer, after lysed at 4°C for 40 minutes centrifuged at 13 000 rpm for 25 minutes, take the supernatant and the Coomassie Brilliant Blue method was used for protein quantification. Mix with 3× Loading Buffer and boil at 95°C for 5 minutes. Then samples were subjected to electrophoresis in 5%, 8%, or 15% SDS‐polypropylene gel at a concentration of 30‐50 µg/lane for 3 hours and then transferred to a nitrocellulose membrane. DYNC1I1 antibody (Abcam 23905 1:2000), β‐actin antibody (Santa sc‐47778 1:1000), TNPO2 antibody (Abcam ab127165 1:1000), SP1 antibody (CST 9389S 1:1000), P300 (CST 86377S 1:1000), HH3 (CST 4499P 1:2000), Ace‐H3K9 (CST 9649 1:1000), Ace‐H3K14 (CST 7627S 1:1000), Ace‐H3K27 (CST 8173 1:1000), HuR (CST 12582 1:1000), CyclineD1 (Santa 753 1:500), CyclineE (Santa 481 1:500), P53 (CST 2527S 1:1000), P21 (Santa 6246 1:500), and CDK2 (Santa 748 1:1000) were added and stored at 4°C overnight. Add horseradish peroxide‐labeled goat anti‐mouse (1:2000) or goat anti‐rabbit (1:2000) secondary antibody for 30 minutes, signal was visualized through a chemiluminescent detection system.

### 2.4 Cell transfection

The gastric cancer cells were inoculated into a six‐well plate at 1.5 × 10^5^/well, after 24 hours, siRNA/shRNA transfection was then carried out by using Lipofectamine 2000 (Invitrogen).Transfection was done according to the manufacturer's instructions. Culture medium was replaced 6‐8 hours later. Cells were continued to be processed at different time points according to the different needs of the experiment.

### 2.5 Cell cycle assay

The effect of TNPO2 on the cell cycle was assessed using propidium iodide staining. Cells were trypsinized after transfection with siTNPO2 for 48 hours, centrifuged at 1000 rpm for 5 minutes, fixed overnight with 75% alcohol precooled to 4°C, then centrifuged at 1000 rpm for 5 minutes, and suspended in PBS (precooled). Centrifugation was again carried out at 4°C, and then 400 μL of PBS containing 50 μg/mL PI and 100 μg/mL RNase was added to the precipitate at 37°C for 30 minutes. After staining, the percentage change of G1, S, and G2 phase cells was evaluated by flow cytometry (BD AccuriTM C6 flow cytometry).

### 2.6 Immunoprecipitation

35 ul Protein G‐sepharose beads (DYNC1I1) and 35 μL Protein A‐sepharose beads (P300, SP1) were added to the EP tube and the beads were washed twice with PBS and then lysis buffer, respectively. The total protein of the control and treatment groups was extracted with lysis buffer, applying Coomassie Bright. The total protein concentration was quantitatively extracted by the method. Forty microliters of protein was taken from each sample as input. The remaining protein lysate and 10 µg of anti‐DYNC1I1 antibody, 10 µg of anti‐P300 antibody (CST 86377S), and 10 µg of anti‐SP1 antibody (CST 9389S) in each sample were then added to Protein G‐sepharose beads and Protein A‐sepharose beads, respectively, and the solution containing beads, protein lysate, and antibody was slowly shaken overnight at 4°C. Then the beads were washed four times with lysis buffer, the supernatant was aspirated, and 2× Buffer 40‐50 µL was added and boiled for 5 minutes, and then the samples were subjected to Western blot analysis.

### 2.7 MTT assay

For MTT assay, gastric cancer cells transfected with TNPO2 siRNA or overexpressed TNPO2 were uniformly tiled in a 96‐well plates (3000 cells per well), 48, 72, and 96 hours later, added MTT (concentration with 5 mg/mL), then continue to incubated for 4 hours. Removed the supernatants, 200 μL DMSO was added to each well and the absorbance was read at 570 nm. Cell viability: relative cell activity = (OD570 measurement − OD570 blank)/(OD570 control − OD570 blank) × 100%.

### 2.8 Cellular apoptosis detection

The Annexin V‐fluorescein isothiocyanate (FITC) Apoptosis Detection kit (BD Biosciences) was used to detect cell apoptosis. Cells were collected 48 hours after transfection with TNPO2 siRNA or over expressed TNPO2. The manufacturer's protocol was followed. Apoptosis changes were then detected using a flow cytometer (BD AccuriTM C6 flow cytometry).

### 2.9 Cytoplasmic and nuclear extraction

Cytoplasmic and nuclear extraction kit for cells purchased from Active Motif. The samples were then subjected to Western blot analysis. Lamin A/C was considered as the internal reference of nuclear protein, and the internal reference of cytoplasmic was used with GAPDH. The cells were collected into a 1.5 mL microcentrifuge tube precooled to 4°C, centrifuged at 500 *g* for 1 minute, the supernatant was discarded, and an appropriate amount of cytoplasmic extraction buffer was added to the cell pellet, vortexed vigorously, and incubated on ice for 30 minutes. Procedures for cytoplasmic and nuclear protein extraction are described in the protocol.

### 2.10 Transcription factor and the binding site prediction

The ALGGEN PROMO software program (http://alggen.lsi.upc.es)^15^ and GeneCards (https://www.genecards.org/)^16^ were used to predict transcription factors. JASPAR (http://jaspar.binf.ku.dk/)^17^ and UCSC website (https://genome.ucsc.edu/)^18^ were used to predict transcription factor binding sites.

### 2.11 Bioinformatics

Involvement of positive correlation genes of KEGG pathway and GO pathway enrichment analysis were evaluated using DAVID online software (https://david.ncifcrf.gov/).^19^ The GEPIA website^20^ was used to predict gene correlation in gastric cancer.

### 2.12 The EdU incorporation assay

The treated gastric cancer cells were seeded into 96‐well plates at a concentration of 2000‐5000 cells/200 µL. After 24 hours of incubation, 50 μmol/L of 5‐ethynyl‐2'‐deoxyuridine (EdU; Ribobio) was added to each well, incubated at 37°C for 2 hours, and then incubated with 4% formaldehyde at room temperature. Fix the cells for 30 minutes. Incubate with 2 mg/mL glycine for 5 minutes. After washing five times with PBS, the cells were reacted with 100 μL of a 1× Apollo reaction mixture for 30 minutes. Then, the nuclei were stained with 1× Hoechst 33342 (5 μg/mL).

### 2.13 Luciferase activity assay

The binding sites on the promoter region of TNPO2 by SP1 were predicted by online data. We construted two plasmids, pGL4.10‐TNPO2 Promoter(Wt, wild type) and pGL4.10‐TNPO2 Promoter(Mut, mutant type). The plasmid was then cotransfected with the reporter plasmid into gastric cancer cells. Determination of luciferase activity was carried out on TECAN Infinite M200Pro reader according to the manufacturer recommendations (Promega); Renilla luciferase was used for normalization.

### 2.14 Statistical analysis

All statistical analyses were carried out using SPSS version 22.0. Differences between groups were compared by using Student's *t* test. Each experiment was repeated three times and the data were expressed as mean + standard deviation. A value of *P* < 0.05 was considered to be statistically significant.

## 3 RESULTS

### 3.1 DYNC1I1 upregulated TNPO2 in gastric cancer cells

**Figure 1:**
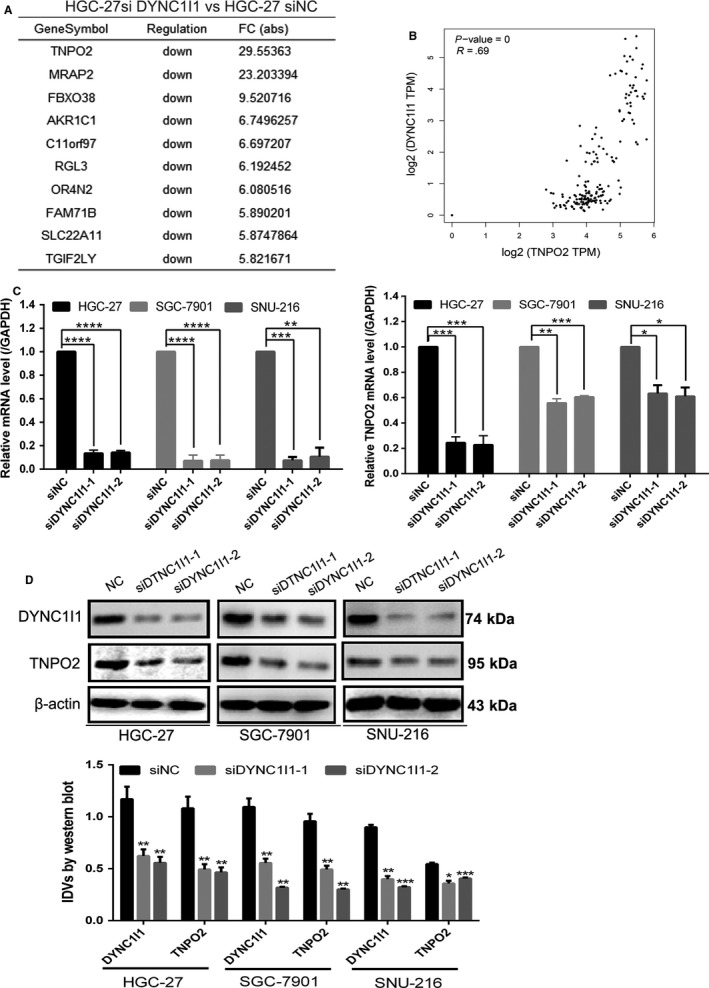
DYNC1I1 upregulated TNPO2 in gastric cancer cell. HGC‐27, SGC‐7901, and SNU‐216 cell lines were transfected with siDYNC1I1 for 48 h. A, After knockdown of DYNC1I1 in HGC‐27 cells, the top 10 upregulated genes reported by microarray genome‐wide expression analysis. B, Correlation between DYNC1I1 and TNPO2 in gastric cancer was performed using GEPIA online platform; differences with *P* < 0.05 were considered statistically significant. C, YNC1I1 and TNPO2 mRNA expression levels were tested by qRT‐PCR in HGC‐27, SGC‐7901, and SNU‐216 cells. D, DYNC1I1 and TNPO2 protein expressions were detected by Western blot analysis in HGC‐27, SGC‐7901, and SNU‐216 cells (*P < 0.05, **P < 0.01, ***P < 0.001, ****P < 0.0001; n = 3, student s *t* test, means ± 95% CI)

Previous studies have confirmed the effect of DYNC1I1 on the biological function of gastric cancer cells. To further explore the mechanism of DYNC1I1 in gastric cancer, affymetrix scanner microarray genome‐wide expression analysis was used after knockdown (KD) of DYNC1I1 in HGC‐27 cells. As shown in Figure 1A, TNPO2 was considered as the primary gene among the downregulated genes. Therefore, the main focus is on TNPO2 in this study. First, to verify the accuracy of the expression profile chip results, the correlation between DYNC1I1 and TNPO2 in gastric cancer was predicted by using the Gene Expression Profiling Interactive Analysis (GEPIA) website. As shown in Figure 1B, DYNC1I1 showed positive correlation with TNPO2 in gastric cancer with a correlation coefficient of 0.69 (*P* < 0.05). Furthermore, RT‐qPCR analysis was performed to detect the changes in TNPO2 mRNA levels after DYNC1I1 knockdown. The results were consistent with those of microarray analysis in HGC‐27 cells (Figure 1C). Western blotting analysis was then performed to detect changes in the relative protein expression levels of TNPO2 after knockdown of DYNC1I1, and this was consistent with mRNA data. The proteins levels of TNPO2 were downregulated after knockdown of DYNC1I1 (Figure 1D). These results indicated that knockdown of DYNC1I1 in gastric cancer cells also downregulated TNPO2, suggesting that DYNC1I1 might be a potential upstream signaling molecule for the activity of TNPO2.

### 3.2 DYNC1I1 upregulated TNPO2 expression by increasing SP1 in gastric cancer cells

**Figure 2:**
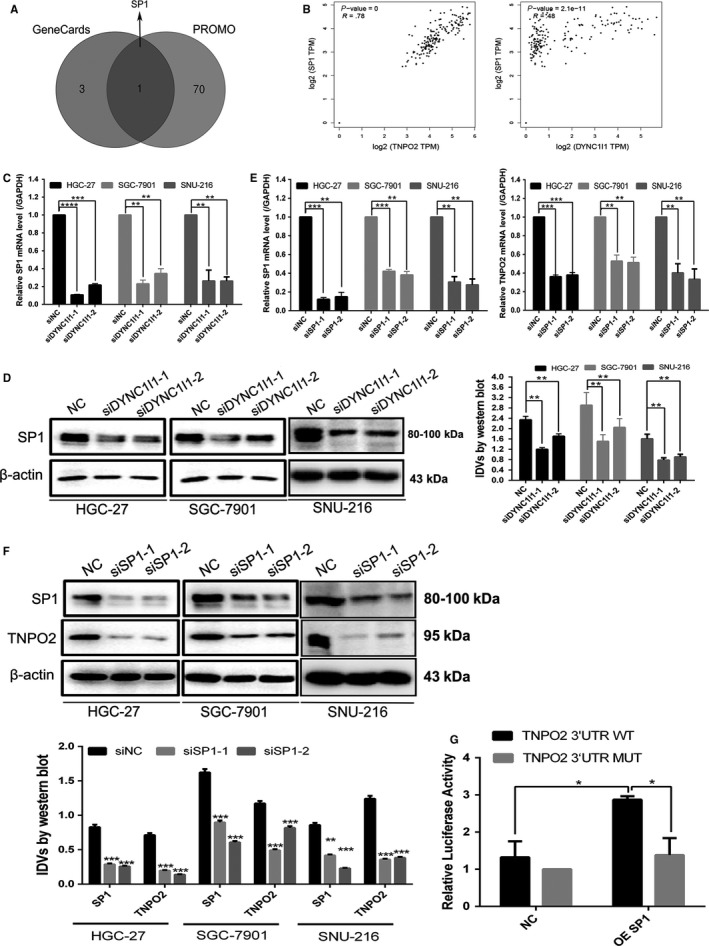
DYNC1I1 upregulated TNPO2 expression by upregulating SP1 in gastric cancer cells. A, Transcription factors (TF) prediction in the promoter region of TNPO2 on GeneCards (only the top four) and ALGGEN PROMO website, respectively, intersect the results obtained by the two websites. B, Correlation between DYNC1I1 and SP1, TNPO2, and SP1 in gastric cancer were performed using GEPIA online platform; differences with *P* < .05 were considered statistically significant. C, SP1 mRNA expression levels were tested by qRT‐PCR in HGC‐27, SGC‐7901, and SNU‐216 cells after transfected with siDYNC1I1 for 48 h. D, SP1 protein expression was detected by Western blot analysis in HGC‐27, SGC‐7901, and SNU‐216 cells after transfected with siDYNC1I1 for 48 h. E, SP1 and TNPO2 mRNA expression levels were tested by qRT‐PCR in HGC‐27, SGC‐7901, and SNU‐216 cells after transfected with siSP1 for 48 h. F, SP1 and TNPO2 protein expressions were detected by Western blot analysis in HGC‐27, SGC‐7901, and SNU‐216 cells after transfected with siSP1 for 48 h. G, The luciferase activities in HGC‐27 cotransfected with SP1 overexpression or NC and luciferase reporters containing TNPO2 promotor WT or TNPO2 promotor MUT. Student's *t* tests were used for statistical analyses (**P < 0.01, ***P < 0.001, ****P < 0.0001; n = 3, student s *t* test, means ± 95% CI)

To further investigate the mechanism by which DYNC1I1 upregulates TNPO2 expression, TNPO2 transcription factor was first predicted by exploring the ALGGEN PROMO website (Figure 2A). At the same time, the TNPO2 transcription factor was predicted on the genecard website. The major four transcription factors were as follows: Arnt, Nkx2‐5, Pax‐6, and SP1. The common transcription factor in both the sites was SP1. It was speculated that DYNC1I1 might regulate the expression of TNPO2 by modulating its transcription factor SP1. Furthermore, the correlation between DYNC1I1 and SP1, as well as TNPO2 and SP1 in gastric cancer was verified by the GEPIA website. As predicted, DYNC1I1 showed positive association with SP1 (correlation coefficient 0.48; *P* < 0.05), and TNPO2 showed positive association with SP1 (correlation coefficient 0.59; *P* < 0.05; Figure 2B). RT‐qPCR and Western blotting analysis were performed to detect SP1 mRNA and protein expression levels after knockdown of DYNC1I1. As shown in Figure 2C, SP1 was downregulated regardless of mRNA or protein levels after knockdown of DYNC1I1. This was consistent with the relationship between DYNC1I1 and SP1 as verified previously by exploring the website. Next, SP1 was knocked down in gastric cancer cells with the aim to detect altered TNPO2 mRNA and protein expression levels. As shown in Figure 2E, it was found that TNPO2 was downregulated in gastric cancer cells at both mRNA and protein expression levels after SP1 knockdown. Dual luciferase assay was then performed to detect whether SP1 binds to TNPO2 promoter site. The results of the assay indicated that luciferase activity was significantly higher when cotransfected with pGL4.10‐TNPO2 promoter (Wt) and SP1 (*P* < 0.05). The luciferase activity in pGL4.10‐TNPO2 promoter (Mu) and pcDNA4.1‐SP1 cotransfection groups was not significantly higher when compared to normal control cells (Figure 2G).These observations demonstrated that DYNC1I1 decreased TNPO2 expression by downregulating SP1 expression in gastric cancer cells, further confirming that SP1 serves as a transcription factor of TNPO2.

### 3.3 SP1 enhanced histone acetylation levels in TNPO2 promoter regions by binding to P300

**Figure 3:**
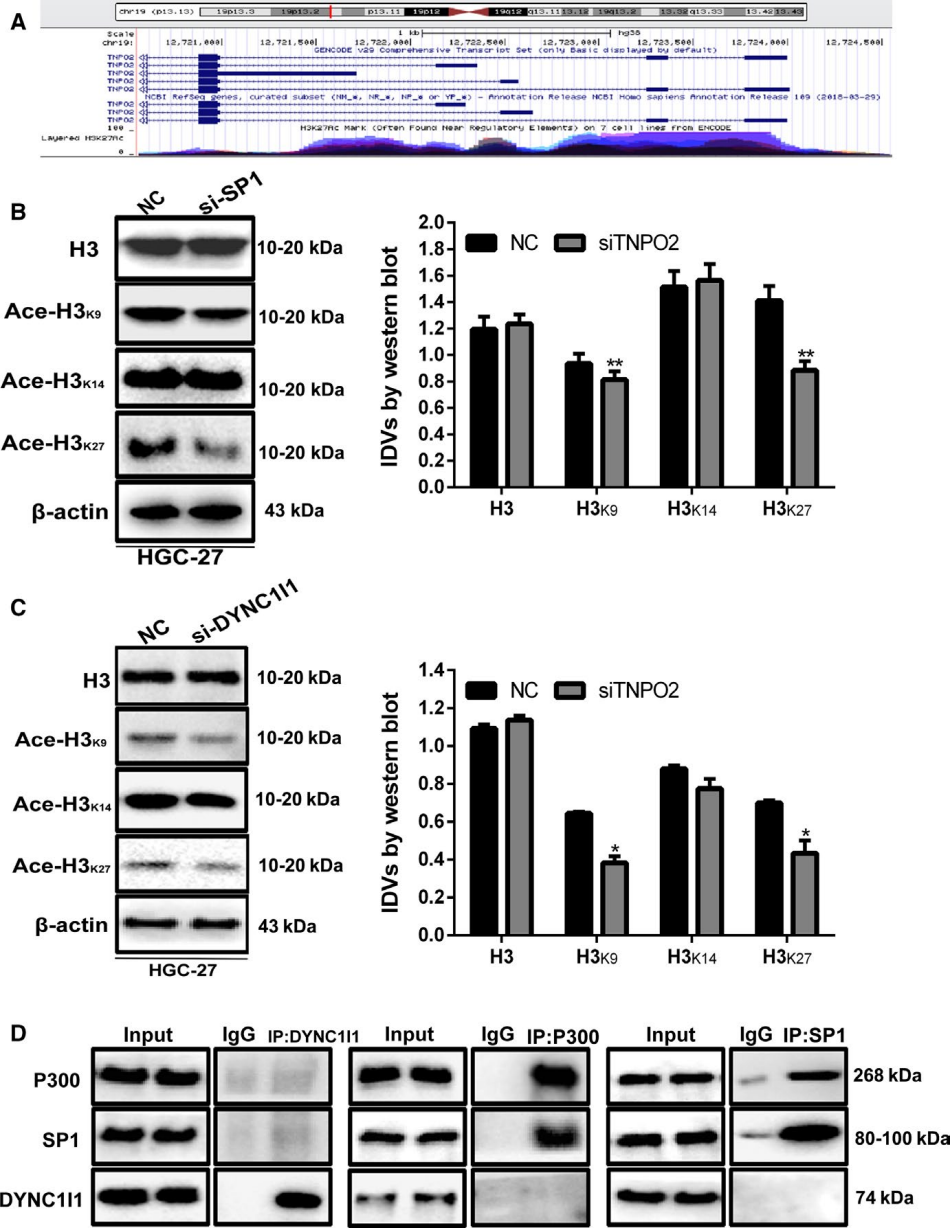
SP1 enhanced the histone acetylation levels in TNPO2 promoter regions by binding to P300. A, The UCSC Genome Bioinformatics Site (http://genome.ucsc.edu/)showed high enrichment of H3K27Ac at the promoter of TNPO2. B and C, Protein expression level of H3, H3_K9_, H3_K14_, and H3_K27_ were detected by Western blot analysis after transfected with siSP1 or transfected with siDYNC1I1 for 48 h. D, Immunoprecipitation assay was performed to test the complex formation of SP1 and P300 physically in HGC‐27 cells (*P < 0.05,**P < 0.01; n = 3, student s *t* test, means ± 95% CI)

Acetylation of H3K27 in TNPO2 promoter region was found by exploring the UCSC website (https://genome.ucsc.edu/) (Figure 3A). Previous studies have shown that SP1 can bind to the acetylation coactivator P300. Coregulation of acetylated target gene promoter region then promotes transcription, and whether similar mechanism exists in this study requires further validation. The level of acetylation in different parts of histone 3 was detected after knockdown of SP1 in HGC‐27 cell. As shown in Figure 3B, H3K9 and H3K27 acetylation levels showed significant downregulation after SP1 knockdown, and both these sites were present at TNPO2 promoter. It was speculated that SP1 affected the levels of TNPO2 promoter acetylation, thus affecting its transcription. Changes in acetylation levels were also found after knockdown of DYNC1I1 in the same cell line (Figure 3C). Next, coimmunoprecipitation assay was performed in HGC‐27 cell to determine whether SP1 binds to P300 or to determine whether DYNC1I1 binds to P300. The results revealed that SP1 can bind to P300 instead of DYNC1I1 (Figure 3D). These results showed that DYNC1I1 regulates SP1 expression in gastric cancer cells, and SP1 not only binds to TNPO2 promoter region but also recruits acetylated coactivator P300 to increase TNPO2 promoter region acetylation, thus driving TNPO2 transcription.

### 3.4 High TNPO2 expression defines a poor prognosis in gastric cancer patients

**Figure 4:**
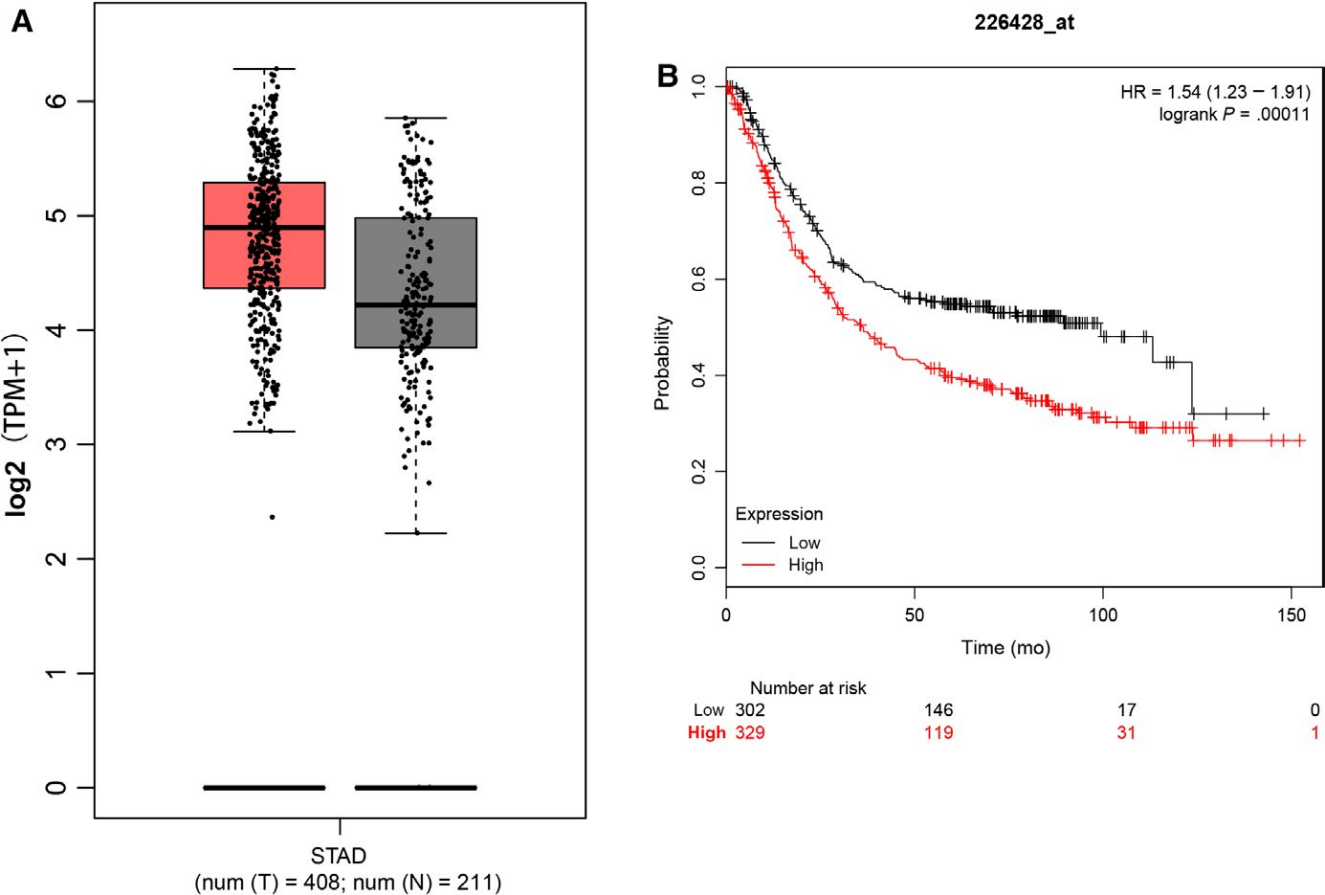
TNPO2 is overexpressed in GC tissues and patients with high TNPO2 expression have a poor prognosis. A, Analysis of TNPO2 expression using GEPIA Website. B, Kaplan‐Meier Plotter analysis of the effect of TNPO2 on GC patients' survival

The role of TNPO2 in gastric cancer has not been studied till date. The expression level of TNPO2 in gastric cancer and adjacent tissues was predicted by using the GEPIA website. As shown in Figure 4A, although no statistical significance was observed, the expression of TNPO2 in gastric cancer was higher than that found in normal tissues. Moreover, the Kaplan‐Meier plotter program (http://kmplot.com/analysis/index.php?p=service%26cancer=gastric)^21^ predicted the effect of TNPO2 on gastric cancer prognosis and the results revealed that high TNPO2 expression showed a lower survival rate in gastric cancer cells (Figure 4B).

### 3.5 TNPO2 promoted gastric cancer cell proliferation and inhibited apoptosis

**Figure 5:**
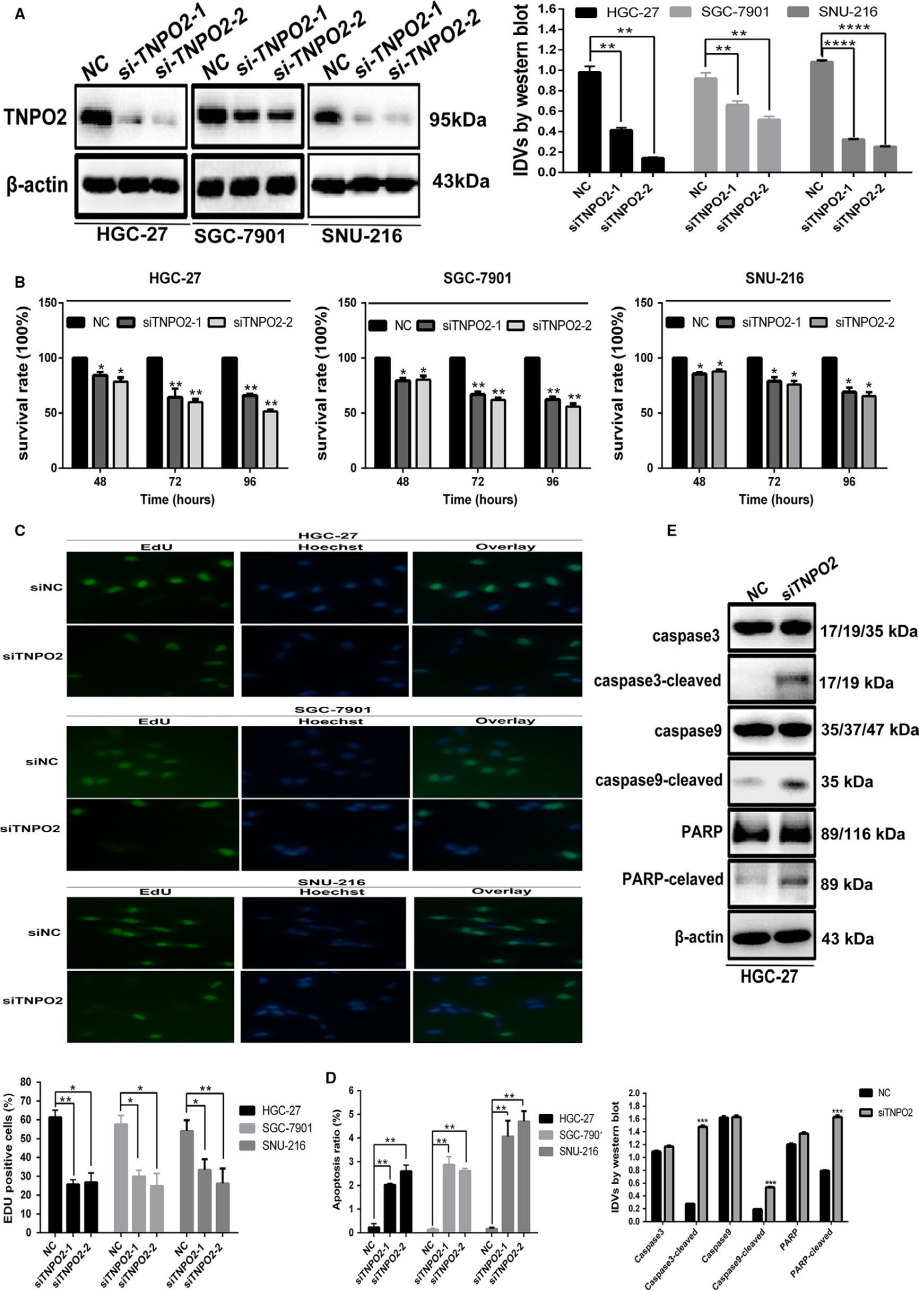
Silencing TNPO2 in gastric cancer cells inhibited proliferation of gastric cancer cells and promoted apoptosis. A, TNPO2 protein expression was detected by Western blot analysis in HGC‐27, SGC‐7901, and SNU‐216 cells after transfected with siTNPO2 for 48 h. B, Analysis of cell proliferation following TNPO2 knockdown in HGC‐27, SGC‐7901, and SNU‐216 cells by MTT assay. C, The apoptotic rates of HGC‐27 and SGC‐7901 cells transfected with siNC or siTNPO2 for 48 h were visualized by flow cytometry. D, EdU incorporation assay. Seventy‐two hours after gastric cancer cells were transfected with siTNPO2 or siNC, followed by incubation with EdU and Hoechst in sequence. Hoechst 33342 (blue) and EdU (green) represent cell nuclei and nuclei of proliferative cells, respectively. The percentages of the EdU‐positive cells are presented (right). E, Apoptotic proteins were detected by Western blot analysis in HGC‐27 cells after transfected with siTNPO2 for 48 h (*P < 0.05, **P < 0.01, ***P < 0.001; n = 3, student s *t* test, means ± 95% CI)

**Figure 6:**
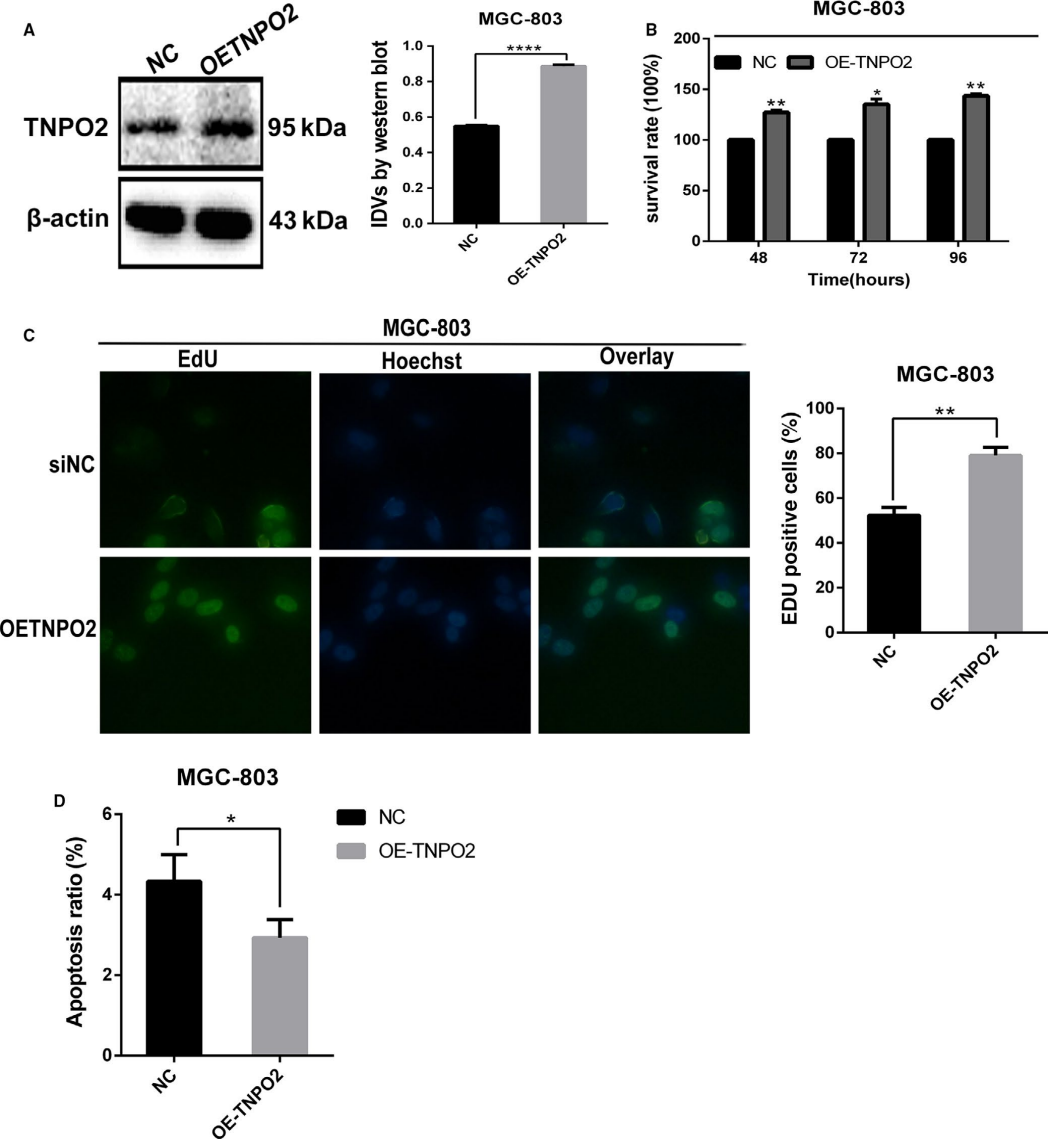
Overexpression of TNPO2 promoted gastric cancer cell proliferation and inhibited apoptosis. A, TNPO2 protein expression was detected by Western blot analysis in MGC‐803 cells after transfected with overexpressing TNPO2 for 48 h. B, MTT shows cell viability of gastric cancer cells after overexpressing TNPO2 for 48, 72, and 96 h. C, The percentages of the EdU‐positive cells after overexpressing TNPO2 for 72 h. D, The apoptotic rates of MGC‐803 cells after overexpressing TNPO2 for 48 h (*P < 0.05, **P < 0.01, ****P < 0.0001; n = 3, student s *t* test, means ± 95% CI)

To assess the biological function of TNPO2 in gastric cancer cells, siRNA was used to transiently knockdown the expression of TNPO2, and Western blotting assay was performed to detect the knockdown efficiency. As shown in Figure 5A, siRNA reduced TNPO2 protein expression. MTT and Edu assays were used to quantify the effect of knockdown of TNPO2 on the ability of cancer cells to proliferate. As shown in Figure 5B, knockdown of TNPO2 reduced the ability of gastric cancer cells to proliferate and increased the inhibition of gastric cancer cell proliferation over time when compared with negative controls. The cell viability of HGC‐27 cells was decreased by about 25% (*P* < 0.05) after 48 hours of knockdown of TNPO2, and by approximately 30% after 72 hours of knockdown of TNPO2 (*P* < 0.05). In addition, the cell viability of HGC‐27 cells was decreased by about 40% after 96 hours of knockdown of TNPO2 (*P* < 0.05). In contrast, SGC‐7901 cell viability was decreased by about 20% (*P* < 0.05) after 48 hours of TNPO2 knockdown, by 30% after 72 hours (*P* < 0.05), and then by 40% after 96 hours (*P* < 0.05). Similar results were obtained after TNPO2 silencing in SNU‐216 cells. As shown in Figure 5C, silencing of TNPO2 inhibited DNA replication in gastric cancer cells. In addition, flow cytometry was performed to detect the changes in the levels of gastric cancer cell apoptosis after 48 hours of transient knockdown of TNPO2 in HGC‐27, SGC‐7901, and SNU‐216 cells. The results revealed that the levels of apoptosis were increased after 48 hours of knockdown of TNPO2 when compared with control group (Figure 5D). Apoptosis‐related proteins were then detected by Western blotting assay (Figure 5E), which showed that the apoptotic proteins caspases 3 and 9 and PARP showed significant increase. For further analyses, overexpression of TNPO2 in MGC‐803 cells and overexpression efficiency were detected by Western blotting (Figure 6A). MTT assay was performed and the results indicated that overexpression of TNPO2 in MGC‐803 cells enhanced the viability of MGC‐803 cells in a time‐dependent manner (Figure 6B). By 48 to 96 hours after overexpression of TNPO2 in MGC‐803 cells, proliferation was increased by approximately 20 to 50% as observed in those without TNPO2 overexpression (*P* < 0.05). Similarly, the EdU results showed that overexpression of TNPO2 in gastric cancer cells increased DNA replication (Figure 6C). Furthermore, the apoptosis levels were decreased after overexpression of TNPO2at 48 hours (Figure 6D). These results showed that TNPO2 promoted gastric cancer cell proliferation and inhibited their apoptosis.

### 3.6 TNPO2 promoted cell proliferation and inhibited apoptosis by upregulating P21

**Figure 7:**
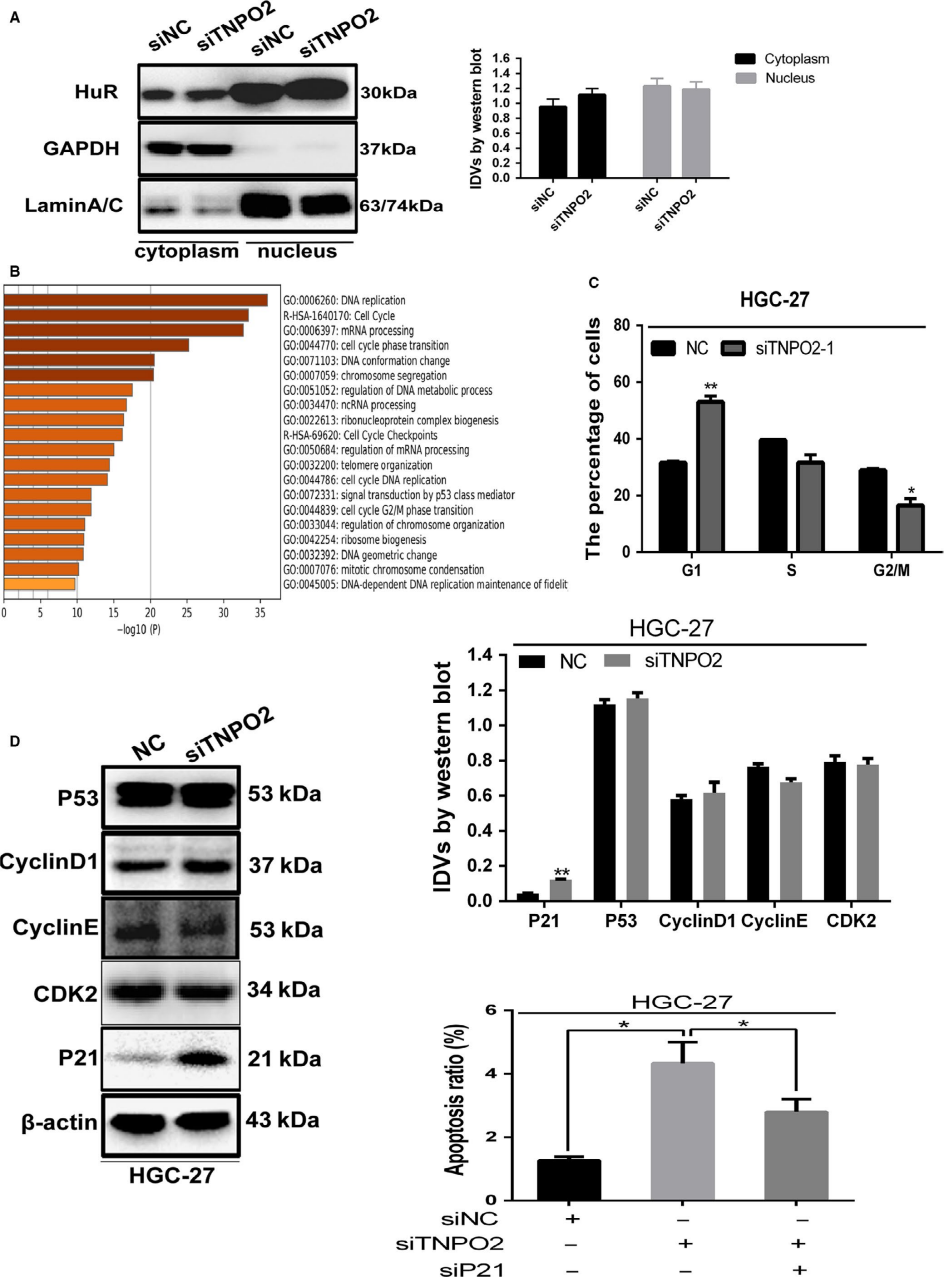
TNPO2 promoted apoptosis and inhibited cell proliferation by upregulated P21. A, HuR protein expression were detected by Western blot analysis in HGC‐27 in cytoplasmic and nuclear extracts, respectively, after transfected with siTNPO2 for 48 h. B, The DAVID website enriches the pathways involved in TNPO2 positively related genes. C, Flow cytometry was performed using PI staining to determine the cell cycle of HGC‐27 cell lines after transfected with siTNPO2 for 48 h. D, P53, CyclinD1, CyclinE, P21, and CKD2 proteins were detected by Western blot analysis in HGC‐27 cells after transfected with siTNPO2 for 48 h. E, The apoptosis rate of cells was detected by flow cytometry after transfection with siNC, siTNPO2, and siP21 for 48 h in HGC‐27 cells (*P < 0.05, **P < 0.01; n = 3, student s *t* test, means ± 95% CI)

To further explore the specific mechanism by which TNPO2 modulates the biological functions in gastric cancer cells, some additional experiments were conducted. Previously it has been reported that TNPO2 promotes Hela cell apoptosis by inhibiting the entry of HuR protein into the nucleus in response to severe stress conditions.^13^ Next, TNPO2 was knocked down in HGC‐27 cells for undergoing nucleoplasmic separation. However, the results showed that the HuR protein showed no significant concentration in the cytoplasm (Figure 7A), suggesting that TNPO2 does not promote apoptosis through this mechanism in gastric cancer cells.

Furthermore, based on the DAVID website, the positive and negative TNPO2 gene pathways were enriched by GO method. The results found that “DNA replication” was the primary pathway observed from both methods, indicating that TNPO2 might regulate the biological behavior of gastric cancer cells through this pathway (Figure 7B). At the same time, the effect of transient TNPO2 knockdown in the cell cycle was detected (Figure 7C). The results showed that the cell cycle was arrested in G1 phase at 48 hours after knockdown of TNPO2 in gastric cancer cell line HGC‐27 [the frequency of G1 phase in the NC interference group was approximately 32%, and the frequency of G1 in TNPO2 siRNA interference group was approximately 56% (*P* < 0.01)]. The G1 phase‐detecting protein was assayed by Western blotting analysis (Figure 7D). The P21 protein was upregulated, and this indicated that P21 might act as a potential downstream mediator of TNPO2. Next, the P21 was silenced in gastric cancer cells and found that P21 silencing in gastric cancer cells partially reversed the increased apoptosis (Figure 7E). These results showed that the potential downstream of TNPO2 might be P21.

## 4 DISCUSSION

TNPO2 is an accessory protein that belongs to the class of RNA‐binding proteins including HuR and hnRNP in the nucleus and belongs to the noncanonical nuclear pathway.^11^, ^12^ It plays an important role as a nuclear accessory protein in normal cells, and at the same time it also plays an important role in tumor cells. In Hela cells, knockdown of TNPO2 can lead to cytoplasmic accumulation of HuR protein, aggravating apoptosis.^13^ Recent studies have shown that TNPO2 is highly expressed in malignant pleural mesothelioma (MPM) tissues as well as cells, and this might formally represent a novel cancer‐specific gene. However, its precise role in MPM has not been studied till now.^14^ In the present study, we found that knockdown of TNPO2 inhibited gastric cancer cell proliferation and promoted their apoptosis.

At present, most of the functional mechanisms of TNPO2 occur due to its role in nuclear import of HuR. TNPO2 promotes muscle fiber formation by influencing the distribution of HuR in cells.^22^ However, in response to severe stress conditions, apoptotic cell death occurs subsequently in tumor cells. The activated caspase cleaves HuR into two fragments, namely HuR‐CP1 and HuR‐CP2. HuR‐CP1 competes with HuR in binding to TNPO2 for HuR accumulation in the cytoplasm, thereby promoting further apoptosis. This suggests that the effect of HuR is accumulated in the cytoplasm after silencing TNPO2 under normal conditions.

In this study, we found that TNPO2 knockdown promoted apoptosis of gastric cancer cells, while this effect was not achieved by abnormal cellular distribution of HuR. This was not completely consistent with the results of the previously published studies, which might be due to the use of different cell lines in experiments. However, it is because of the affect of cycle‐associated protein P21.

The initial function of P21 is that it acts as a mediator in cellular senescence via p53‐dependent and independent pathways.^23^, ^24^ On one hand, senescence can also be mediated through reactive oxygen species (ROS)‐based mechanisms,^25^ and on the other hand, p21 is currently considered as a potent universal cyclin‐dependent kinase (CDK) inhibitor,^26^, ^27^ serving as an important regulator of cell cycle progression in G1 as well as S phases.^28^, ^29^ In the current study, the cell cycle was arrested in G1 phase following TNPO2 knockdown in gastric cancer cells, while the expression of P21 was upregulated. However, CyclinD1, CyclinE, CKD2, and P53 showed no significant changes.

Unfortunately, the precise mechanism of action of P21 has not been clarified yet. Whether to weaken the binding stability of CDK2 and cyclinE or CDK4/6 and CyclinD1 by inhibiting its catalytic activity or by other unknown mechanisms remains to be clarified. In the aspect of P21 and its role in apoptosis, the current study found that P21 has two sides. First, p21 plays a role in apoptosis inhibition, and by inhibiting the initiation of apoptotic caspase cleavage cascade by tumor necrosis factor‐related apoptosis‐inducing ligand (DR4/TRAIL) receptor. This in turn inhibits apoptosis and provides survival advantages of human tumor cells.^30^ Second, it is known that the functional expression of p21 has protective effects on tumor cell apoptosis by inducing cell growth arrest or cellular senescence.^31^ In contrast, the activated caspase cleaves P21 to P15 to promote cellular apoptosis. We further found that TNPO2 knockdown promoted gastric cancer cell apoptosis, and enhanced the functional expression of caspases 3 and 9, and P21 in this study, which indicated that activated P21 does not inhibit the caspase activity. It is speculated that activated caspase activity cleaves P21 in order to promote apoptosis.

The specific mechanism of DYNC1I1 regulation of TNPO2 was further explored and the results revealed that SP1 plays an important role in it. The transcription factor SP1 is a basal transcription factor that regulates the so‐called housekeeping genes.^32^ SP1 also showed overexpression in many cancer types and its high expression showed association with poor prognosis.^33^, ^34^, ^35^, ^36^ In addition to direct binding to DNA, SP1 can simultaneously recruit P300. Functional activation of P300 serves as a transcriptional coactivator that does not directly bind to DNA, catalyzing the promoter‐bound histone acetylation, and leading to gene activation.^37^, ^38^, ^39^ For the first time we found that DYNC1I1 decreased TNPO2 expression by downregulating SP1. In addition to its role as a TNPO2 transcription factor, SP1 acetylates TNPO2 promoter region histone 3, which in turn is mediated by recruiting P300 by SP1. However, DYNC1I1 cannot be combined with P300.

## 5 CONCLUSIONS

In conclusion, we found that DYNC1I1 upregulates TNPO2 expression, and increases the expression of transcription factor SP1. Activated SP1 not only binds to TNPO2 promoter site but also recruits P300 for acetylation of TNPO2 promoter site histones to promote TNPO2 transcription. The role of TNPO2 as a nuclear accessory protein in tumors remained unclear. TNPO2 promoted gastric cancer cell proliferation and inhibited apoptosis, and this effect might be achieved by downstream engagement of P21. Whether TNPO2 exerts this effect on P21 is unclear and requires research regarding the transportation of substances into the nucleus and on to which substances. Hence, further research is warranted to clarify the specific mechanism. By investigating the underlying mechanism of DYNC1I1 and TNPO2 in gastric cancer, the molecular markers that help in predicting the prognosis of gastric cancer were screened. For more precise treatment of gastric cancer, these potential molecular targets are intended.

## CONFLICT OF INTEREST

The authors declare that they have no competing interests.

## AUTHOR CONTRIBUTIONS

Xiujuan Qu, Zhi Li, and Yunpeng Liu conceived and designed the experiments. Libao Gong, Yizhe Wang, and Jin Wang performed the experiments. Libao Gong and Yizhe Wang analyzed the data. Libao Gong, Xiujuan Qu, and Yunpeng Liu wrote the paper.

## Data Availability

All data generated or analyzed during this study are included in this published article.
